# Caries Rates in Different School Environments Among Older Adolescents: A Cross-Sectional Study in Northeast Germany †

**DOI:** 10.3390/children12081014

**Published:** 2025-08-01

**Authors:** Ahmad Al Masri, Christian H. Splieth, Christiane Pink, Shereen Younus, Mohammad Alkilzy, Annina Vielhauer, Maria Abdin, Roger Basner, Mhd Said Mourad

**Affiliations:** 1 Department of Pediatric Dentistry, University Medicine Greifswald, 17489 Greifswald, Germany; splieth@uni-greifswald.de (C.H.S.); shereen.younus@stud.uni-greifswald.de (S.Y.); alkilzym@uni-greifswald.de (M.A.); annina.vielhauer@uni-greifswald.de (A.V.); maria.abdin@uni-greifswald.de (M.A.); r.basner@gmx.net (R.B.); mhd.mourad@uni-greifswald.de (M.S.M.); 2 Department of Orthodontics, University Medicine Greifswald, 17475 Greifswald, Germany; 3 Department of Restorative Dentistry, Periodontology and Endodontology, University Medicine Greifswald, 17475 Greifswald, Germany; christiane.pink@uni-greifswald.de

**Keywords:** adolescents, dental caries, oral epidemiology, oral health, social inequalities

## Abstract

Background/Objectives: Educational background is an aspect of socio-economic status, that may be associated with higher caries risk. This study aimed to investigate differences in caries prevalence between different school types for older adolescents in Greifswald, Germany. Methods: Cross-sectional data were collected as part of compulsory dental school examinations between 2020 and 2023. Oral health status was assessed according to WHO criteria by six calibrated examiners and reported as mean D_3_MFT (D_3_: dentin caries, M: missing, F: filled, SD/±: standard deviation). To compare educational backgrounds, the adolescents were divided into two groups according to their age and type of school (11–15 and 16–18 years old). Results: The study included 5816 adolescents (48.7% females) with a mean D_3_MFT of 0.65 (Q1–Q3: 0–1); 73.8% were clinically caries-free, having D_3_MFT = 0, confirming the polarization in caries experience with 2.5 ± 2.13 SaC index. The logistic regression model showed a significantly increased Odds Ratio for having caries in relation to age, being male, having plaque or gingivitis (*p* < 0.005). There were significant differences in caries experience and prevalence between school types, where high schools had the lowest D_3_MFT values in both age groups (0.39 ± 1.17 and 0.64 ± 1.49, respectively). The highest D_3_MFT values were in schools for special educational needs in younger adolescents (1.12 ± 1.9) and in vocational schools in older adolescents (1.63 ± 2.55). Conclusions: In a low-caries-risk population, there were significant differences in caries experience and prevalence among adolescents in different school types. Prevention strategies should aim to reduce the polarization in caries across different educational backgrounds in late adolescence.

## 1. Introduction

Despite the substantial decline in caries prevalence among children and adolescents in numerous countries around the world over recent decades, the latest studies report around 2.3 billion untreated caries in permanent teeth globally [1], making oral caries a global yet neglected critical public health challenge. Reviews also report that 46.2% of children worldwide have caries in their primary teeth (n = 80,405), while more than half of the children worldwide (n = 53.8%) have caries in their permanent teeth (n = 1,454,871) [2]. The exact prevalence of caries in children could be underestimated, as many countries do not have epidemiological data or established community oral care programs.

In Germany, the most recent epidemiological studies report a reduction of 89.7% in caries among 12-year-old schoolchildren, with the mean DMFT dropping from 4.9 in 1989–1992 to an impressive 0.5 in 2023 [3]. Despite this reduction, the polarization in caries experience is alarming. On one hand, 77.6% of 12-year-olds have no caries experience, while on the other hand, the remaining 22.4% have positive caries experience with a mean DMFT of 2.4. With such polarization, further prevention measures at the community level for the whole population would not bring any benefit to high-caries-risk individuals, as these measures might not reach them. Therefore, individual measures should be adopted, which start with identifying the high-caries-risk individuals. While risk factors for early childhood caries are well reported in multiple systematic reviews, making the identification of high caries-risk individuals possible [4], reports regarding the risk factors in older adolescents are less decisive, with more focus on dietary habits than social or environmental factors [5]. Therefore, the relationship of social factors with caries in older adolescents should be investigated.

As social inequalities are observed even in developed countries, and considering that different school types and education systems represent different socio-economic classes, this study focuses on identifying the types of schools and education systems that are attended by adolescents with higher caries experience. As this proportion of individuals have increasing DMFT with age, leading to more treatment need after the complete eruption of all the permanent teeth and into adulthood, this study aims to report the caries rates of the older 11–18-year-old adolescents in schools in the region of Greifswald in Northeast Germany in different school environments, which might aid in targeting them for individual preventive and educational measures before reaching adulthood, where they will be more difficult to detect.

Moreover, schools offer a unique and standardized setting for accessing large and diverse adolescent populations. Given that dental school examinations are mandatory in Germany, they provide an ideal platform for epidemiological surveillance and the identification of high-risk groups. While many national and international studies focus on children up to the age of 12, older adolescents are often overlooked despite undergoing significant lifestyle changes that can affect their oral health. This study addresses this gap by examining older adolescents across different educational settings, which can support future public health strategies to further reduce and prevent oral caries.

## 2. Materials and Methods

This study includes data collected through the compulsory school dental examinations conducted by the health authorities in Vorpommern-Greifswald municipality in the state of Mecklenburg-Pomerania. With over 237,000 inhabitants [6], Vorpommern-Greifswald municipality is one of the largest regions of its state, which is administratively further divided into 3 districts (Greifswald, Anklam and Pasewalk). This study was mainly concerned with the districts of Greifswald and Anklam, as very few dental examinations were conducted in Pasewalk district in the same of period of time due to difficulties from the COVID-19 pandemic.

### 2.1. Data Collection

All the included data were collected by the health authorities between 1 October 2020 to 31 April 2023. As the dental examinations were compulsory, no inclusion or exclusion criteria were applied. All the parents/guardians were informed about the date of the dental examinations through the school administration. Depending on the total number of children in each school, the examinations were planned on multiple days within one or two weeks. Only children absent from their school on all the examination days were not examined. The dental examinations were performed in all school types with children aged 11 to 18 starting from the sixth grade until the twelfth grade, including the following school types:

- Regional school (“Regionale Schule”): A type of secondary school including grades 5 to 10. Its certificate allows either joining a higher school or vocational school.
- Comprehensive school (“Gesamtschule”): A type of school including all learning tracks and integrating all academic levels, including a proportion of children with mental or physical disabilities.
- High school (“Gymnasium”): An academically demanding secondary school preparing the students for university education, including grades 5–12.
- School for special educational needs (“Förderschule”): Schools especially designed for children with learning disabilities, speech and language challenges, or developmental delays. It promotes the individual needs of the children and has different categorizations of the grades based on performance not age.
- Vocational school (“Berufsschule”): A type of dual education system combining theoretical learning with practical job training, which is usually between 2 and 4 years after the age of 15.

With the inclusion of all these types of schools, almost all adolescents 11–18 years old were included, as adolescents are regulated by law to visit one type of these schools at least until the age of 15, with the possibility to afterwards either proceed with education in university with a high school certificate “Abitur” or start a job training (e.g., in vocational schools).

### 2.2. Clinical Examination

The routine dental school examinations are performed in a non-dental setting with the dentist sitting on a normal chair and the student sitting on a normal chair opposite to the dentist, typically involving examining the mouth (soft and hard tissues) using an intraoral mirror and either an external light or headlamp. A probe is not commonly employed but is available if food debris obstructs the visual assessment. If needed, cotton swabs can be used to dry the teeth for better assessment. The teeth are examined with the registration of any abnormalities in teeth number, size or form. A total of six examiners, working as dentists in the Department of Pediatric Dentistry at the University of Greifswald, conducted the school examinations. They were trained using an online platform designed for epidemiological examinations in Germany [7]. The training included two modules of theoretical and practical instruction using clinical images, followed by a third module for actual calibration, which could be repeated with a random order and the selection of other cases if the initial calibration was unsuccessful. The calibration was considered successful if a kappa value of at least 0.65 was achieved.

Dental caries were assessed using the WHO’s criteria with consideration of the ICDAS criteria [8] and registered in a special program to facilitate school medical examinations called ISGA [9]. The program includes general characteristics for each child, including date of birth, date of examination, sex, the name of the school, and the grade. Moreover, the presence of visual plaque and gingivitis on the upper front teeth was assessed by a simple yes or no through the dentist at the beginning of the examination. For each tooth, one of the following codes should be used for the documentation:

- S: The tooth is healthy
- U: The tooth is not yet assessable (unerupted)
- D: Dental caries with cavity (ICDAS 3 and above)
- Z: Tooth destructed due to caries (tooth not restorable)
- F: Filled tooth (or covered with a crown)
- M: Extracted tooth due to caries
- Y: Extracted tooth due to orthodontic reasons
- I: Initial caries (ICDAS 1 or 2)
- H: Hypoplasia (including molar incisor hypomineralization)
- V: Fissure sealant

In cases of untreated caries (D or Z), the child is given a short letter explaining the need for dental treatments and recommending a visit to the dentist. The registration of the tooth status and the results of the dental examinations are entered by one of three trained dental nurses. The final data can be pseudonym exported as an excel sheet for statistical analysis.

### 2.3. Statistical Analysis

The statistical analyses were performed with software SPSS version 30.0.0.0 (IBM©). Statistically significant results were defined as two-tailed *p*-values less than 0.05. *p*-value corrections were not implemented due to the exploratory nature of this study.

D_3_MFT was calculated by summing the teeth with the assessments D, Z, F, and M and reported as the mean value with standard deviations. The individuals with D_3_MFT = 0 were considered “caries free”, and the D_3_T value was calculated to measure treatment need, while the Care Index was measured as the proportion of treated caries FT and MT in relation to the total number of affected teeth to assess dental treatments. The Specific affected Caries Index (SaC) was measured by calculating the mean D_3_MFT of only the individuals with a positive caries experience (D_3_MFT > 0) to consider the polarization in caries experience in low-caries-risk communities [10]. Further analysis of the individuals with caries experience included D_3_T and D_3_MFT for individuals with untreated caries (D_3_T > 0). The general characteristics of the examined adolescents as well as the mean values of the above-mentioned caries assessments were reported as frequencies and means with standard deviations. A logistic regression model was used to calculate the Odds Ratio (OR) for the prevalence of dental caries (having D_3_MFT > 0), considering available variables such as age, sex, plaque, gingivitis, and type of school.

Since education in schools in the German system is compulsory until the ninth grade (ca. 15 years old), adolescents would mostly move to a different school after the ninth grade depending on their future plans to either pursue a high school certificate and possibly a university degree or attend vocational school with practical training. However, some school types such as high schools or comprehensive schools might also have younger adolescents between the fifth and ninth grade. For this reason, and in order to assess the differences between the different school environments regarding caries experience, the adolescents were divided into two groups, with the cut point set as the tenth grade (15 years old). Therefore, the first group represented the adolescents from the regular sixth grade or its equivalents until the tenth grade (regardless of their age, as some schools for special educational needs may have older adolescents in lower grades), including regional schools, comprehensive schools, high schools and schools for special educational needs, while the other group represented eleventh and twelfth grades (16–18 years old) in high schools, comprehensive schools, and vocational schools. For each of these groups, frequencies and means were reported and compared, and a logistic regression model was used again to calculate the Odds Ratio (OR) for the prevalence of dental caries (having D_3_MFT > 0), considering the same available set of variables.

### 2.4. Ethical Aspects

The data extracted from the ISGA software (Version 5) program were exported in a pseudonymized format. For the final analysis, all findings were anonymized. This study adheres to the ethical guidelines for medical research involving human subjects as outlined in the Declaration of Helsinki in 1964 and its subsequent revisions, and it received approval from the University of Greifswald’s ethics committee (Reg. No.: BB48/10a). Since the dental examinations were mandatory as regulated by the law of the schools in the state of MV, obtaining additional written informed consent for the dental examination was not required. As regulated in the German school law of the state of Mecklenburg-Vorpommern, the parents/guardians of the school attendants are to be informed on the date and the process of the examinations a couple of days ahead. Before performing the dental examination, the process and aim of it is explained for the students, who give a verbal consent to the process. In case of the unwillingness to perform the examination, the case was reported to the supervising health authorities but no action was undertaken in the school.

## 3. Results

The total number of examined adolescents in the Vorpommern-Greifswald municipality between 2020 and 2023 included in this study was 5816 (48.7% females). The overall results confirmed the low caries risk of adolescents in Germany, with 73.8% of the adolescents having a D_3_MFT score of zero, which is clinically caries-free. The median D_3_MFT was 0 (percentiles Q1–Q3: 0–1). Although the mean D_3_MFT score of 0.65 ± 1.55 can be considered low, the SaC index showed a mean D_3_MFT score of 2.5 ± 2.13, reflecting the polarization in caries experience in this low-caries-risk population. The overall results of caries experience according to age is shown in Table 1, while Table 2 includes further analysis regarding dental treatments (Care Index) and dental treatment need (D_3_T), particularly among adolescents with caries experience, to illustrate the degree of polarization.

**Figure 1:**
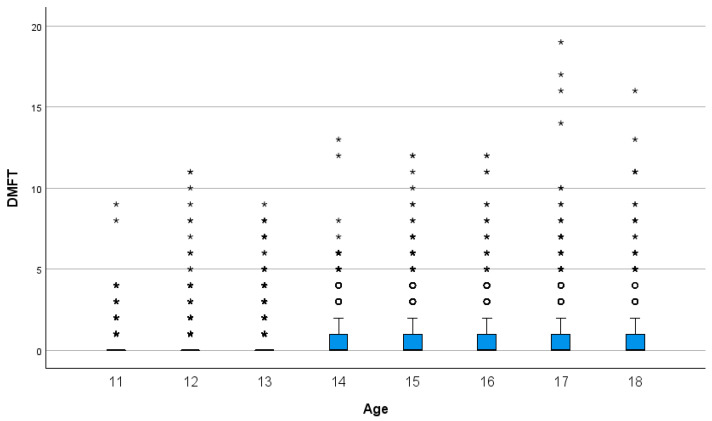
A box plot showing the mean DMFT according to the age in the study population (n = 5816). Boxes represent the interquartile range, the line indicates the median. ○ are mild outliers. * are extreme outliers.

Figure 1 shows the box plots of DMFT according to age, which confirms the increase in DMFT with age and the polarization of caries experience.

A logistic regression model was performed to determine the Odds Ratio (OR) for a D_3_MFT > 0, including all collected variables, which statistically confirmed the increased risk of caries with age, being male, having plaque or gingivitis, and attending a school for special educational needs (Table 3).

### 3.1. Results of the Adolescents of the Eleventh and Twelfth Grades (16–18 Years Old)

The total number of adolescents examined in the eleventh and twelfth grades was 791 (17.6% in comprehensive schools, 51.7% in high schools, and 30.7% in vocational schools). Table 4 shows the differences in categorical and numerical variables between the adolescents in the different school environments, with no significant differences in sex, plaque or gingivitis, whereas D_3_MFT showed significant differences between all school types in all its components.

A logistic regression model was used to calculate the OR of having D_3_MFT > 0 among older adolescents in the eleventh and twelfth grades (16–18 years old), which revealed that being in vocational school was the only statistically significant factor leading to increased risk of caries in these age groups (Table 5).

### 3.2. Results of the Adolescents up to the Tenth Grade (11–15 Years Old)

The total number of the examined adolescents between the regular sixth grade and the tenth grade in all school types was 5024 (38% in regional schools, 23.8% in comprehensive schools, 32.4% in high schools and 5.8% in schools for special educational needs). There were statistically significant differences in all available variables between the adolescents in different school environments, as shown in Table 6.

Analogous to the analysis of the group of older adolescents, a logistic regression model was performed to assess the OR of having caries, which revealed a statistically significant increase in the OR with age, being male, having plaque or gingivitis, and attending school for special educational needs, whereas attending high school or comprehensive school showed significantly lower ORs (Table 7).

### 3.3. Differences in D_3_MFT Between the Different School Types

**Figure 2:**
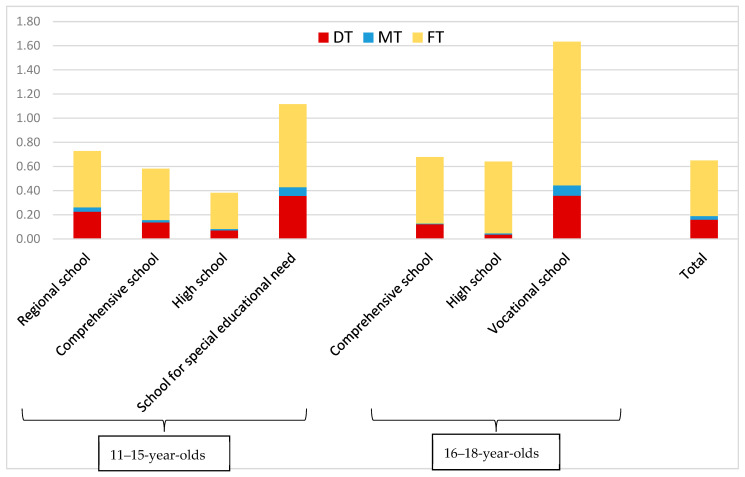
Mean D_3_MFT scores and their components in the different school types and age groups (n = 5816).

The D_3_MFT scores of the study population categorized according to the school type and age are presented in Figure 2, with clearly higher mean D_3_MFT scores in schools for special educational needs for adolescents until the eleventh grade, and even higher scores for adolescents in the eleventh and twelfth grades in vocational schools (16–18 years old).

## 4. Discussion

The main aim of this study was to report and analyze the caries experience of adolescents (11–18 years old) in the region of Greifswald in Northeast Germany. With 73.8% of the study population having D_3_MFT = 0 and a mean D_3_MFT of 0.65 ± 1.55, the overall results confirm the general low caries risk of the study population, which is consistent with national reports from epidemiological studies reporting low caries experience in children and adults in the state of MV and Germany [3,7]. Unlike the situation in young children in the same region, where there is a clear dental care problem represented by a Care Index of 26.1% in three year olds and a large proportion of untreated dental caries, with 26.9% of the six years olds having d_3_t > 0, indicating the need for more dental treatments [11], the Care Index in adolescents was at lowest 61.8% by the 11-year-olds and reached 83.2% by the 18-year-olds. However, the SaC index, which calculates the caries level in children with caries experience (D_3_MFT > 0), showed a mean of 2.5 D_3_MFT and 0.62 D_3_T. A closer look at the individuals with untreated caries (D_3_T > 0) revealed a mean D_3_MFT of 2.97 and a mean D_3_T of 1.89. These findings highlight a significant issue not only in caries prevention for these individuals but also in dental care. As these individuals are less dependent on their parents or guardians than younger children are, the problem of untreated caries may not be due to neglect but rather due to dental fear or financial hesitation to visit the dentist, although national health insurance in Germany would cover all basic dental treatments [12].

The polarization in caries experience of the study population reflected by the 2.5 SaC index in a low caries-risk population, with 73.8% having no caries experience, complies with the polarization throughout Germany in children and adolescents as reported in national epidemiological studies [3,7], which also is similar to the polarization of caries in adolescents in Spain and Denmark [13,14]. In order to further investigate the characteristics and factors leading to an increased risk of having caries in our polarized low-caries-risk population, a logistic regression model was performed and showed a higher OR for a positive D_3_MFT score increasing with age, which agrees with multiple studies investigating the correlation between DMFT and age in adolescents in Iran, Croatia, and Uganda [15,16,17]. Regarding the differences in caries risk between males and females, some studies show a general higher risk for females [18], while the national results of caries experience of the 12-year-olds in Germany report higher risk for males, similar to our results [3]. However, some studies showed no differences in sex regarding caries experience [19,20]. These inconsistencies may reflect variations in cultural, geographic, or age-related factors, suggesting that sex alone may not be a reliable predictor of caries risk. The regression model also revealed a logical increase in the OR with the visual presence of plaque or gingivitis. While this agrees with studies regarding the correlation of gingivitis and presence or progression of caries, these studies could not find such a correlation with plaque [21,22]. This can be explained by the ease of plaque removal before examinations, especially if the examinations are performed in clinical visits, while our examinations were in the daily routine of school, where the children probably did not perform extra comprehensive toothbrushing prior.

While the correlation of socio-economic class and caries experience is well reported in the literature [23,24,25], it is neither feasible nor reliable to perform a valid assessment of socio-economic class in regular compulsory dental examinations. In our study, we assume that the different school types represent differences in socio-economic class, as some schools or education systems require the payment of extra fees or the consistent attention of the parents, which represents a higher socio-economic class, with our results confirming that children in these schools had the lowest caries risk compared to other school types, similar to another study in Saudi Arabia [26]. With regional schools serving as the reference group, comprehensive schools and high schools showed a significantly lower OR for having caries, while schools for special educational needs had a significantly higher OR. These results were also confirmed when performing the regression analysis specifically for adolescents up to the tenth grade without the 16–18 years old adolescents in the eleventh and twelfth grades. These results agree with earlier studies in younger adolescents in Germany, where differences in caries experience were observed between children in different school types and education systems [27,28].

The logistic regression model for the older adolescents including vocational schools and excluding younger adolescents between the sixth and tenth grades revealed no significant OR for the included factors. The only exception was the significant OR for individuals in vocational school compared to those in comprehensive or high school, which highlights the much higher caries risk of these individuals. These results confirm the results shown in the descriptive analysis, where less than half of the adolescents in vocational schools (49.4%) were “caries free” compared to 70% in comprehensive schools and 74.8% in high schools in the same age groups. Only two studies in the literature were found to report the oral health of 18-year-olds in vocational schools in Poland, reporting high treatment need in these individuals [29,30].

As Germany is one of the few countries where regular caries prevention twice a year and basic dental treatments are fully covered by statutory health insurance, further prevention measures at the community level are unlikely to yield substantial benefits in caries reduction, and the focus should be turned towards individual prophylaxis of those with higher caries risk. The first step towards further measures in caries prevention would be the identification of these high-risk individuals. Considering all the performed analyses and the results, our data confirmed the polarization in caries experience in a low-caries-risk population and highlighted the characteristics leading to a higher risk of having caries in adolescents. The presence of plaque or gingivitis increases the risk of caries but is not a unique indicator, as it merely reflects the situation of oral hygiene in the very short term before the examination, which might change from one week to the other. Although being male was statistically associated with a higher risk of caries, it is not a sufficient factor to warrant sex-specific preventive measures. The most important outcome of our results is the higher caries risk of the individuals in schools for special educational needs and vocational schools, which can easily be targeted for intensive prevention measures as well as for education on oral health and dental care. The differences in the general characteristics and in caries experience, as well as caries risk between the school types, reflect the socio-economic differences and inequalities in children, as reported in other countries such as Italy, Sweden, and the USA [31,32,33]. Our results showed great similarities in the general characteristics and in the caries experience to the results of national epidemiological examinations of 12-year-olds in Germany [3,7]. As these examinations did not include adolescents above the age of 12, our study had the aim to further detect the trends in caries experience and the higher caries risk in these individuals. As reported in our results, a clear higher risk of having caries is observed among students of schools for special educational needs and vocational schools. Any further efforts to optimize the prevention of dental caries should target individuals of these school types, which have lower socio-economic class, when they are still accessible for preventive measures and before they reach the age of 18, when outreach becomes more difficult.

This study has multiple limitations that need to be considered in the interpretation of its results. Although the overall results confirm the very low caries experience of the study population, the examination was not performed in controlled dental settings, potentially leading to missing some composite fillings and falsely categorizing some affected teeth as healthy. Moreover, the lack of radiographic examination may lead to underestimation of the true prevalence of caries in cases of approximal lesions or the presence of composite fillings underneath a fissure sealant. However, the overall results of national examinations on adults in dental settings does not show a great jump in the caries experience, which shows that the proportion of missed carious lesions in these examinations is marginal. Another limitation of this study is the assessment of socio-economic class only according to school and education type. However, including questionnaires with data collection is not a part of the compulsory examination and would have required a separate informed consent, which in turn jeopardizes the representativity of the population and increases the risk of selection bias. On the other hand, this study has multiple strengths, increasing its value. The efforts to include all individuals and all school types in the target study population resulted in an exceptionally large sample size. Furthermore, the number of examinations that were calibrated minimizes the risk of individual errors by data collection. The last aspect is the inclusion of vocational schools in the recruitment, which are never considered as regular schools or included in national epidemiological examinations in Germany, with very scarce studies regarding the oral health of these individuals in the literature.

## 5. Conclusions

In a low caries-risk adolescence population, the polarization in caries experience necessitates targeting higher-risk individuals for further improvements in caries prevention. Higher caries experience and prevalence was found among adolescents attending vocational schools and schools for special educational needs. Efforts should be made to reach these high-risk individuals in their schools for preventive and educational measures before reaching adulthood, where they become harder to reach.

## Figures and Tables

**Table 1:** Overall results of caries experience of the study population according to age.

Age Group	N	Female	DMFT = 0	Mean D_3_MFT	Mean ID_3_MFT	Mean D_3_T
11	633 (10.9)	328 (51.8)	536 (84.7)	0.28 ± 0.85	1.09 ± 1.73	0.11 ± 0.58
12	917 (15.8)	431 (47)	724 (79.0)	0.46 ± 1.22	1.22 ± 2.02	0.16 ± 0.79
13	1018 (17.5)	519 (51)	796 (78.2)	0.48 ± 1.20	1.48 ± 2.32	0.15 ± 0.68
14	962 (16.5)	440 (45.7)	704 (73.2)	0.6 ± 1.34	1.89 ± 2.81	0.17 ± 0.62
15	808 (13.9)	387 (47.9)	559 (69.2)	0.81 ± 1.67	2.15 ± 3.07	0.15 ± 0.64
16	660 (11.3)	308 (46.7)	460 (69.7)	0.8 ± 1.70	2.27 ± 3.39	0.15 ± 0.58
17	508 (8.7)	266 (52.4)	324 (63.8)	1.1 ± 2.26	2.78 ± 3.74	0.24 ± 1.05
18	310 (5.3)	153 (49.4)	189 (61.0)	1.25 ± 2.39	3.27 ± 4.12	0.21 ± 0.81
Total	5816 (100)	2832 (48.7)	4292 (73.8)	0.65 ± 1.55	1.85 ± 2.88	0.16 ± 0.71

Data are presented as number (percentage) or mean ± SD.

**Table 2:** Dental treatment need and Care Index of the study population according to age.

Age Group	SaC Index ^a^	Mean DT If D_3_MFT > 0	N if D_3_T > 0 (%)	Mean D_3_MFT If D_3_T > 0 (SD)	Mean D_3_T If D_3_T > 0 (SD)	Care Index ^b^
11	1.84 ± 1.36	0.7 ± 1.35	40 (6.3)	2.13 ± 1.77	1.7 ± 1.65	61.8
12	2.21 ± 1.80	0.78 ± 1.57	72 (7.9)	2.78 ± 2.50	2.1 ± 1.96	64.6
13	2.2 ± 1.67	0.7 ± 1.31	84 (8.3)	2.57 ± 2.12	1.86 ± 1.55	68.0
14	2.24 ± 1.75	0.64 ± 1.06	97 (10.1)	2.65 ± 1.95	1.7 ± 1.08	71.4
15	2.64 ± 2.07	0.5 ± 1.08	69 (8.5)	3.29 ± 2.57	1.81 ± 1.36	80.8
16	2.66 ± 2.15	0.49 ± 0.97	59 (8.9)	2.88 ± 2.35	1.66 ± 1.11	81.5
17	3.05 ± 2.86	0.67 ± 1.66	47 (9.3)	4.28 ± 3.66	2.62 ± 2.40	77.9
18	3.2 ± 2.91	0.54 ± 1.23	34 (11.0)	4.03 ± 3.56	1.91 ± 1.66	83.2
Total	2.5 ± 2.13	0.62 ± 1.28	502 (8.6)	2.97 ± 2.56	1.89 ± 1.60	75.0

Data are presented as number (percentage) or mean ± SD. ^a^ SaC: mean D_3_MFT if D_3_MFT > 0. ^b^ Care Index: MT + FT/DMFT in %.

**Table 3:** Logistic regression model for DMFT > 0 for the whole study population (n = 5816).

	OR	95% CI		*p*
		Lower	Upper	
Age, years	1.261	1.217	1.307	<0.001
Sex, male	1.278	1.129	1.446	<0.001
Plaque, yes	1.291	1.095	1.522	0.002
Gingivitis, yes	1.240	1.048	1.468	0.012
Regional school	1-Reference			
Comprehensive school	0.737	0.625	0.868	<0.001
High school	0.423	0.359	0.499	<0.001
Vocational school	1.058	0.778	1.439	0.719
School for special educational needs	1.592	1.227	2.067	<0.001

**Table 4:** Differences between adolescents of the eleventh and twelfth grades (16–18 years old) in the different school types (n = 791).

Category	Variables	Comprehensive School	High School	Vocational School	Total	*p*-Value
Sex	Male	67 (48.2)	179 (43.8)	120 (49.4)	366 (46.3)	0.335 ^a^
	Female	72 (51.8)	230 (56.2)	123 (50.6)	425 (53.7)	
Plaque	Yes	48 (34.5)	121 (29.6)	92 (37.9)	261 (33)	0.086 ^a^
	No	91 (65.5)	288 (70.4)	151 (62.1)	530 (67)	
Gingivitis	Yes	68 (48.9)	185 (45.2)	120 (49.4)	373 (47.2)	0.531 ^a^
	No	71 (51.1)	224 (54.8)	123 (50.6)	418 (52.8)	
D_3_MFT	D_3_MFT = 0	97 (69.8)	306 (74.8)	120 (49.4)	523 (66.1)	<0.001 ^a^
	D_3_MFT > 0	42 (30.2)	103 (25.2)	123 (50.6)	268 (33.9)	
Age		17.38 ± 0.65	17.8 ± 0.52	17.82 ± 0.73	17.73 ± 0.63	<0.001 ^b^
D_3_T		0.12 ± 0.44	0.04 ± 0.23	0.36 ± 1.13	0.15 ± 0.69	<0.001 ^c^
MT		0.01 ± 0.08	0.01 ± 0.12	0.09 ± 0.41	0.03 ± 0.25	<0.001 ^c^
FT		0.55 ± 1.74	0.59 ± 1.42	1.19 ± 2.02	0.77 ± 1.7	<0.001 ^c^
D_3_MFT		0.68 ± 191	0.64 ± 1.49	1.63 ± 2.55	0.95 ± 1.99	<0.001 ^c^
ID_3_MFT		2.32 ± 2.91	1.67 ± 2.44	4.37 ± 4.81	2.61 ± 3.61	<0.001 ^c^

The data are presented as n (%) or as mean values ± standard deviation. ^a^ Chi-square test. ^b^ One way ANOVA. ^c^ Kruskal–Wallis-Test.

**Table 5:** Logistic regression model for D_3_MFT > 0 of adolescents in the eleventh and twelfth grades (16–18 years old).

	OR	95% CI		*p*
		Lower	Upper	
Comprehensive School	1-Reference			
High School	0.746	0.480	1.160	0.193
Vocational School	2.251	1.427	3.551	<0.001
Sex, male	1.308	0.955	1.792	0.095
Plaque, yes	0.659	0.430	1.010	0.055
Gingivitis, yes	0.912	0.605	1.375	0.660
Age, years	1.129	0.883	1.444	0.333

**Table 6:** Differences between adolescents (11–15 years old) in the different schools (n = 5024).

Category	Variables	Regional School	Comprehensive School	High School	School for Special Educational Needs	Total	*p*-Value
Sex	Male	1028 (53.9)	612 (51.2)	785 (48.2)	193 (66.3)	2618 (52.1)	<0.001 ^a^
	Female	880 (46.1)	584 (48.8)	844 (51.8)	98 (33.7)	2406 (47.9)	
Plaque	Yes	984 (51.6)	603 (50.4)	664 (40.8)	176 (60.5)	2427 (48.3)	<0.001 ^a^
	No	924 (48.4)	593 (49.6)	965 (59.2)	115 (39.5)	2597 (51.7)	
Gingivitis	Yes	1186 (62.2)	700 (58.5)	874 (53.7)	206 (70.8)	2966 (59)	<0.001 ^a^
	No	722 (37.8)	496 (41.5)	755 (46.3)	85 (29.2)	2058 (41)	
D_3_MFT	D_3_MFT = 0	1362 (71.4)	903 (75.5)	1330 (81.6)	173 (59.5)	3768 (75)	<0.001 ^a^
	D_3_MFT > 0	546 (28.6)	293 (24.5)	299 (18.4)	118 (40.5)	1256 (25)	
Age		13.62 ± 1.63	13.74 ± 1.63	14.68 ± 1.39	13.96 ± 1.73	14.01 ± 1.63	<0.001 ^b^
D_3_T		0.23 ± 0.84	0.14 ± 0.59	0.07 ± 0.49	0.36 ± 0.96	0.17 ± 0.71	<0.001 ^b^
MT		0.04 ± 0.26	0.02 ± 0.17	0.01 ± 0.13	0.07 ± 0.36	0.03 ± 0.22	<0.001 ^b^
FT		0.47 ± 1.14	0.43 ± 1.22	0.30 ± 0.97	0.69 ± 1.48	0.42 ± 1.14	<0.001 ^b^
D_3_MFT		0.73 ± 1.57	0.58 ± 1.46	0.39 ± 1.17	1.12 ± 1.9	0.61 ± 1.46	<0.001 ^b^
ID_3_MFT		1.89 ± 2.82	1.63 ± 2.55	1.42 ± 2.57	2.93 ± 3.36	1.74 ± 2.73	<0.001 ^b^

Data are presented as n (%) or as mean values ± standard deviation. ^a^ Chi-square test. ^b^ Kruskal–Wallis-Test.

**Table 7:** Logistic regression model for DMFT > 0 of adolescents between the sixth and tenth grades (11–15 years old).

	OR	95% CI		*p*
		Lower	Upper	
Regional school	1-Reference			
Comprehensive school	0.777	0.656	0.921	0.004
High school	0.430	0.363	0.510	<0.001
School for special educational needs	1.583	1.218	2.059	<0.001
Sex, male	1.287	1.124	1.474	<0.001
Plaque, yes	1.233	1.031	1.475	0.022
Gingivitis, yes	1.275	1.059	1.535	0.010
Age, years	1.317	1.263	1.374	<0.001

## Data Availability

Restrictions apply to the availability of these data. Data were pseudonymously obtained from the local health authorities of Vorpommern-Greifswald in Mecklenburg-Vorpommern, Germany, and are not available publicly due to data protection reasons.
